# Clinical relevance of procalcitonin and C-reactive protein as infection markers in renal impairment: a cross-sectional study

**DOI:** 10.1186/s13054-014-0640-8

**Published:** 2014-11-19

**Authors:** Ji Hyeon Park, Do Hee Kim, Hye Ryoun Jang, Min-Ji Kim, Sin-Ho Jung, Jung Eun Lee, Wooseong Huh, Yoon-Goo Kim, Dae Joong Kim, Ha Young Oh

**Affiliations:** Division of Nephrology, Department of Medicine, Samsung Medical Center, Sungkyunkwan University School of Medicine, 81 Irwon-ro, Gangnam-gu, Seoul, 135-710 Republic of Korea; Biostatistics and clinical epidemiology center, Samsung Medical Center, Sungkyunkwan University School of Medicine, Seoul, Korea

## Abstract

**Introduction:**

Although the clinical application of procalcitonin (PCT) as an infection marker in patients with impaired renal function (estimated glomerular filtration rate (eGFR) <60 ml/min/1.73 m^2^) has been increasing recently, it is unclear whether PCT is more accurate than C-reactive protein (CRP). We investigated the clinical value of CRP and PCT based on renal function.

**Methods:**

From November 2008 to July 2011, a total of 493 patients who simultaneously underwent CRP and PCT tests were enrolled. The area under the receiver operating characteristic (ROC) curve and characteristics of both markers were analyzed according to infection severity and renal function.

**Results:**

In patients with impaired renal function, the area under the ROC curve was 0.876 for CRP and 0.876 for PCT. In patients with infection, CRP levels differed depending on whether the infection was localized, septic, or severely septic, whereas PCT levels were higher in patients with severe sepsis or septic shock. In patients without infection, CRP did not correlate with eGFR, while PCT was negatively correlated with eGFR.

**Conclusion:**

This study demonstrates that CRP is accurate for predicting infection in patients with impaired renal function. The study suggests that in spite of its higher cost, PCT is not superior to CRP as an infection marker in terms of diagnostic value.

## Introduction

Severe infection and sepsis with accompanying dysfunction or failure of multiple organs are major causes of morbidity and mortality in patients with chronic kidney disease (CKD) [1,2]. These patients are particularly susceptible to infection because of functional deterioration of many components of the immune system [3]. However, it is difficult to differentiate between infectious causes and non-infectious causes of systemic inflammatory responses because of chronic elevation of inflammatory markers and nonspecific clinical symptoms that are common among patients with CKD [4,5]. In particular, the specificity of C-reactive protein (CRP) has been of concern because CRP has been found to be nonspecifically elevated in chronic inflammatory conditions, such as atherosclerosis and CKD [5,6].

Recently, procalcitonin (PCT) has become widely used for differentiating between infectious and non-infectious disease. Some studies have found that PCT is a more accurate marker for predicting infection than CRP in patients with impaired renal function as well as in those with normal renal function [7-9]. However, the diagnostic value or the best cutoff value of PCT in patients with impaired renal function or renal replacement therapy (RRT) remains unknown because the elimination route of PCT has not been described yet. Additionally, previous studies evaluating PCT in patients with impaired renal function included either an unrepresentative study sample or a very small sample size. Although a recent meta-analysis study reported that both CRP and PCT have acceptable specificity in diagnosing infection in patients with impaired renal function, that meta-analysis included studies with high heterogeneity in the study population [10], thus, there are still debates over the relative advantages of PCT compared to CRP, especially regarding diagnostic accuracy even with its higher cost [10,11]. The aim of this study is to evaluate the clinical relevance of PCT compared to CRP as an infection marker based on difference in renal function.

## Materials and methods

### Study population

We performed a cross-sectional study to evaluate the diagnostic value of PCT compared to CRP in regard to renal function after obtaining approval from the Institutional Review Board (IRB) at Samsung Medical Center in compliance with the Declaration of Helsinki (IRB approval number: 2013-06-020). As we collected data retrospectively, patient consent was not applicable. Study participants were selected from the patient population at Samsung Medical Center, a 2,000-bed tertiary referral center in Seoul, Korea. Eligible participants were adults (≥18 years old) with suspected infection who underwent CRP and PCT tests simultaneously (within a 6-hour period) between 1 November 2008 and 31 July 2011. Patients were enrolled regardless of the hospitalization setting, such as ward, intensive care unit, or emergency room, to include patients with variable disease severity.

The total number of patients who received CRP and PCT tests within a 6-hour period upon admission during the study period was 25,075. We recruited 23,819 of these eligible patients and then randomly selected 100 patients from each class of renal function based on estimated glomerular filtration rate (eGFR) to enroll patients evenly according to renal function (class 1: eGFR ≥90 mL/min/1.73 m^2^; class 2: eGFR 60 to 90 mL/min/1.73 m^2^; class 3: eGFR 30 to 60 mL/min/1.73 m^2^, class 4: eGFR 15 to 30 mL/min/1.73 m^2^; class 5: eGFR ≤15 mL/min/1.73 m^2^ without RRT; class 6: eGFR ≤15 mL/min/1.73 m^2^ with hemodialysis (HD); or class 7: eGFR ≤15 mL/min/1.73 m^2^ with peritoneal dialysis (PD)). After excluding patients with an unclear clinical course of infection, a total of 493 patients were ultimately enrolled in this study.

The randomly selected patients were divided into two groups based on their infection status as determined by a review of their medical records. Patients were defined as having an infection when a clinically definable source of infection was present, as confirmed by microbiology tests and/or positive blood cultures. In cases of suspected PD peritonitis, a diagnosis was made if the peritosol white blood cell count was greater than 100 cells/mm^3^ and the percentage of neutrophils was greater than 50%, even if no microbe was identified [12]. Cases with clinical ambiguities or obscurities that made it difficult to classify infection status were excluded. Thus, cases with no microbiologically-confirmed culture results to accompany a clinical diagnosis of infection were excluded. Likewise, cases with microbiologically-confirmed culture results but no clinical diagnosis of infection were also excluded. Additionally, cases without a conclusive clinical diagnosis of systemic inflammatory response or organ failure were excluded from both groups.

Important clinical and laboratory variables, such as age, sex, preexisting underlying disease, clinical diagnosis, severity of infectious condition, microbiology of culture from infection source, hematologic data, and chemistry data, were collected when CRP and PCT tests were conducted. The American College of Chest Physicians/Society of Critical Care Medicine Consensus Conference definition of sepsis stage was used to classify the severity of infection as sepsis, severe sepsis, or septic shock [13].

The primary outcome was a comparison between the reliability of CRP and PCT as diagnostic markers of infection in patients with impaired renal function. The secondary outcomes included the best cutoff value of CRP and PCT levels in patients with impaired renal function, the association between CRP and PCT levels and infection severity in patients with impaired renal function, and the association between CRP and PCT levels and renal function in the absence of infection.

### Measurements

Renal function was assessed based on eGFR levels using the modification of diet in renal disease (MDRD) equation. Serum CRP concentrations were measured with an immunoturbidimetric assay (CRPL3, Roche Diagnostics, Indianapolis, IN, USA) and the lower reference limit was 0.3 mg/dL. Serum PCT concentrations were measured with an enzyme-linked fluorescent assay (Brahms Diagnostica GmbH, Berlin, Germany) and the lower reference limit was 0.05 ng/mL.

### Statistical analyses

For statistical analyses, SPSS PAWS version 20.0 (SPSS Inc., Chicago, IL, USA) was used. For continuous variables, data are shown as the median and IQR if the data were not normally distributed. Categorical data are shown as the number and percentage. Continuous variables were analyzed using the Mann-Whitney *U-*test. The Kruskal-Wallis test and Tukey test using ranks for post-hoc comparison were used for multiple comparisons. Categorical data were analyzed using the chi-square test. To compare the predictive ability of CRP and PCT for infection, receiver operating characteristic (ROC) curves and the areas under the respective curve (AUC) were calculated. AUCs were compared using the nonparametric approach of DeLong *et al*. for two correlated AUCs [14]. Spearman correlation analysis was used for nonparametric data. *P*-values less than 0.05 were considered statistically significant.

## Results

Among the 700 patients assessed, 207 patients were excluded because of clinical ambiguities about their infection status, as described in Materials and methods. A total of 493 patients were finally analyzed. Patients were divided into two groups based on renal function (group I: eGFR ≥60 mL/min/1.73 m^2^; group II: eGFR <60 mL/min/1.73 m^2^). In group I, 73 patients (48%) had an infection and 78 patients (52%) did not have an infection, and in group II, 186 patients (46%) had an infection and 156 patients (54%) did not have an infection.

Baseline characteristics are summarized in Table 1. There were no significant differences in age, sex, or major comorbidities (such as diabetes mellitus (DM) or hypertension) and renal function based on infection status (Table 1). Disease categories are shown in Table 2. The most common infectious disease in the sample population was urinary tract infection (26.6%), followed by intra-abdominal infection (17.0%) (Table 2). The most common non-infectious disease was cardiovascular disease (26.1%) (Table 2).

**Table 1:** **Baseline characteristics**

	**Total**			**Group I**			**Group II**		
	**Infection-**	**Infection+**	***P***	**Infection-**	**Infection+**	***P***	**Infection-**	**Infection+**	***P***
Number	234	259	-	78	73	-	156	186	-
Age, yr	61 (24)	62 (20)	0.84	52 (22)	51 (32)	0.9	69 (24)	66 (18)	0.49
Female, %	90 (38.5)	115 (44.4)	0.20	30 (38.5)	33 (45.2)	0.83	60 (38.5)	82 (44.1)	0.64
DM, %	68 (29.1)	85 (32.8)	0.38	7 (9.0)	16 (21.9)	0.08	61 (39.1)	69 (37.1)	0.9
HTN, %	104 (44.4)	117 (45.2)	0.9	15 (19.2)	19 (26.0)	0.67	89 (57.1)	98 (52.7)	0.89
eGFR, mL/min/1.73 m^2^	29.9 (68.8)	22.2 (63.0)	0.13	104.6 (59.6)	100.2 (38.1)	0.9	14.4 (20.3)	14.4 (25.3)	0.78

Categorical data were analyzed using the chi-square test and continuous variables were analyzed using the Mann-Whitney *U-*test. Data represent median (IQR) or number (percentage). Group I: eGFR ≥60 mL/min/1.73 m^2^, Group II: eGFR <60 mL/min/1.73 m^2^. DM, diabetes mellitus; HTN, hypertension; eGFR, estimated glomerular filtration rate.

**Table 2:** **Disease categories**

**Disease category**	**Number (%)**
**Disease categories in patients with infection**	
Urinary tract infection	69 (26.6)
Intra-abdominal infection	44 (17.0)
Pneumonia	39 (15.1)
Peritonitis	29 (11.2)
Bacteremia with unknown focus	27 (10.4)
Skin and soft tissue infection	26 (10.0)
Vascular infection	13 (5.0)
Catheter related infection	8 (3.1)
Others	4 (1.5)
Total	259 (100)
**Disease categories in patients without infection**	
Cardiovascular disease (atrial fibrillation, myocardial infarction, heart failure)	61 (26.1)
Renal disease (rhabdomyolysis, thrombotic thrombocytopenic purpura-hemolytic uremic syndrome)	35 (15.0)
Gastroenterohepatic disease (gastrointestinal bleeding, toxic hepatitis)	34 (14.5)
Hemato-oncologic disease (lymphoma B symptom, sarcoidosis)	30 (12.8)
Respiratory disease (chronic obstructive pulmonary disease, interstitial lung disease)	19 (8.1)
Rheumatologic disease (gout, adult onset Still's disease)	15 (6.4)
Neuropsychiatric disease (cerebral infarction, intracranial hemorrhage)	13 (5.6)
Endocrinological disease (adrenal insufficiency, hypoglycemia, diabetic ketoacidosis)	8 (3.4)
Musculoskeletal disease (fractures)	4 (1.7)
Others (drug fever, pain shock)	15 (6.4)
Total	234 (100)

The median values of CRP and PCT are shown in Table 3. The median values of CRP and PCT were significantly higher in patients with infectious diseases (CRP: *P* <0.001; PCT: *P* <0.001). Sub-analyses of CRP and PCT based on renal function showed that both markers were significantly elevated in patients with infection (Table 3).

**Table 3:** **C-reactive protein (CRP) and procalcitonin (PCT) levels by renal function and infection status**

	**CRP, mg/dL**			**PCT, ng/mL**		
	**Infection-**	**Infection+**	***P***	**Infection-**	**Infection+**	***P***
Total	1.12 (3.63)	10.62 (17.94)	<0.001	0.15 (0.42)	2.86 (22.73)	<0.001
Group I (eGFR ≥60)	2.38 (8.46)	8.42 (10.82)	<0.001	0.05 (0.13)	0.45 (4.21)	<0.001
Group II (eGFR <60)	0.83 (2.48)	12.08 (20.02)	<0.001	0.25 (0.53)	4.76 (32.37)	<0.001
eGFR ≥90	2.38 (5.36)	6.09 (10.36)	0.001	0.05 (0.10)	0.21 (1.98)	<0.001
60≤ eGFR <90	2.55 (11.45)	11.06 (16.52)	0.03	0.07 (0.20)	1.28 (20.37)	<0.001
30≤ eGFR <60	0.96 (2.37)	12.19 (16.46)	<0.001	0.09 (0.22)	2.88 (19.80)	<0.001
15≤ eGFR <30	0.75 (2.28)	19.86 (21.69)	<0.001	0.17 (0.42)	23.22 (77.22)	<0.001
eGFR <15						
Without RRT	0.62 (2.65)	18.82 (16.54)	<0.001	0.25 (0.53)	14.44 (65.76)	<0.001
HD	1.89 (3.53)	10.19 (22.12)	<0.001	0.55 (0.80)	7.37 (36.34)	<0.001
PD	0.40 (1.33)	3.30 (9.78)	<0.001	0.40 (0.45)	1.31 (10.74)	0.001

Statistics were analyzed by the Mann–Whitney *U-*test. Data represent median (IQR). Group I: eGFR ≥60 mL/min/1.73 m^2^, Group II: eGFR <60 mL/min/1.73 m^2^. eGFR, estimated glomerular filtration rate; RRT, renal replacement therapy; HD, hemodialysis; PD, peritoneal dialysis.

**Figure 1:**
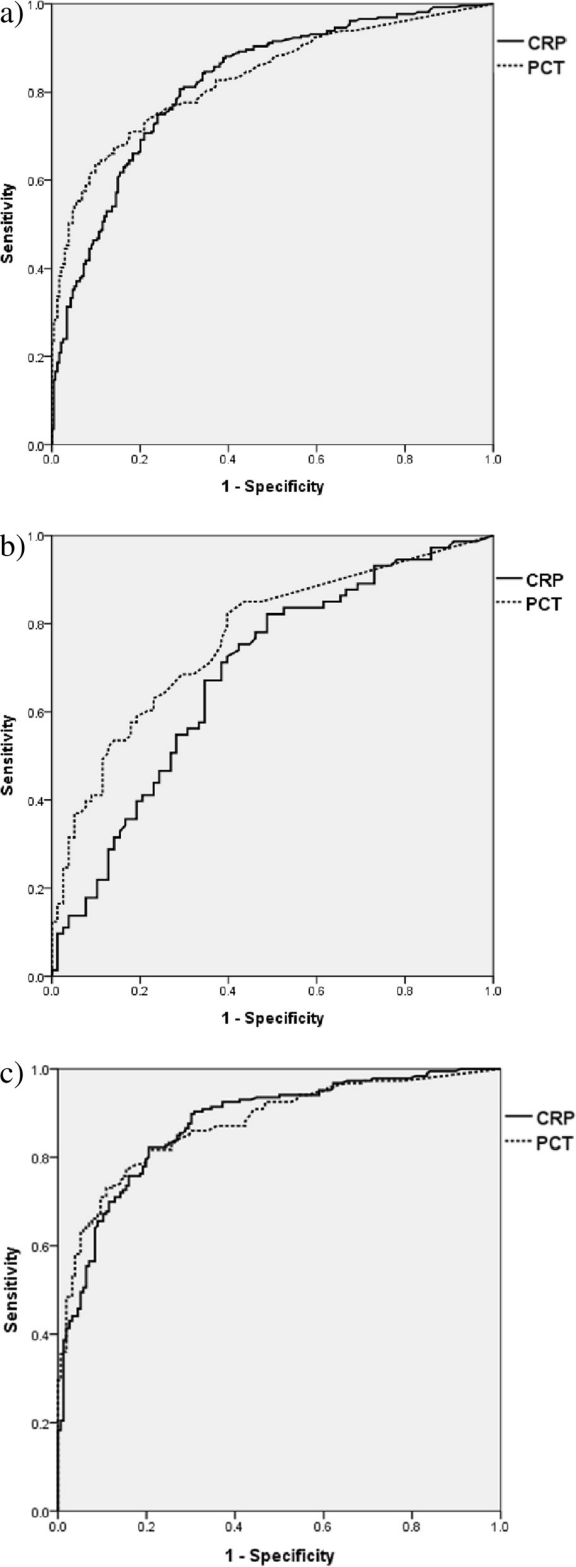
**Receiver operating characteristic curves of C-reactive protein (CRP) and procalcitonin (PCT) for predicting infection. CRP is shown as solid lines, PCT as broken lines. (a)** Receiver operating characteristic (ROC) curves for predicting infection in all patients. CRP: area under the curve (AUC) 0.819 (95% CI 0.782, 0.856), best cutoff value 3.08 mg/dL; sensitivity 81%, specificity 71%; positive predictive value (PPV) 0.75, negative predictive value (NPV) 0.77. PCT: AUC, and best cutoff value 0.831 (95% CI 0.795, 0.866) and 1.1 ng/mL, respectively; sensitivity and specificity 64% and 90%, respectively; PPV and NPV 0.88 and 0.69, respectively. **(b)** ROC curves for prediction of infection in patients with normal renal function (group I). CRP: AUC, and best cutoff value 0.684 (95% CI 0.587, 0.782) and 2.49 mg/dL, respectively; sensitivity and specificity 82% and 51%, respectively; PPV and NPV 0.61 and 0.75, respectively. PCT: AUC, and best cutoff value 0.766 (95% CI 0.681, 0.851) and 0.08 ng/mL, respectively; sensitivity and specificity 82% and 60%, respectively; PPV and NPV 0.66 and 0.78, respectively. **(c)** ROC curves for prediction of infection in patients with impaired renal function. CRP: AUC, and best cutoff value 0.876 (95% CI 0.839, 0.912) and 3.08 mg/dL, respectively; sensitivity and specificity 82% and 79%, respectively; PPV and NPV 0.83 and 0.79, respectively. PCT: AUC, and best cutoff value 0.876 (95% CI 0.839, 0.912) and 1.1 ng/mL, respectively; sensitivity and specificity 73% and 89%, respectively; PPV and NPV 0.89 and 0.74, respectively.

The diagnostic value of CRP and PCT for predicting infection was compared using the ROC curve. The AUC for the diagnosis of infection versus non-infection was 0.819 (95% CI 0.782, 0.856) for CRP and 0.831 (95% CI 0.795, 0.866) for PCT (Figure 1a). For group I, the AUC was 0.684 (95% CI 0.587, 0.782) for CRP and 0.766 (95% CI 0.681, 0.851) for PCT (Figure 1b). For group II, the AUC was 0.876 (95% CI 0.839, 0.912) for CRP and 0.876 (95% CI 0.839, 0.912) for PCT (Figure 1c). There were no differences between the AUCs of CRP and PCT in the two groups (total: *P* = 0.59 for all patients; *P* = 0.12 for group I; *P* = 0.9 for group II). When the AUC of CRP and PCT was analyzed further in patients with systemic inflammatory response syndrome (SIRS) (excluding patients without SIRS from both groups), CRP still showed no inferiority to PCT in differentiating infection. In patients with SIRS, the AUC of CRP was 0.804 (95% CI 0.759, 0.850) and that of PCT was 0.802 (95% CI 0.757, 0.847). In SIRS patients with normal renal function, the AUC of CRP was 0.629 (95% CI 0.532, 0.725) and that of PCT was 0.694 (95% CI 0.603, 0.785). In SIRS patients with impaired renal function, the AUC of CRP was 0.890 (95% CI 0.849, 0.931) and that of PCT was 0.858 (95% CI 0.811, 0.906). CRP was not inferior to PCT in patients with SIRS.

Additionally, to evaluate whether the diagnostic accuracy could be increased using CRP and PCT together, the AUC of both CRP and PCT together was compared with the AUC of CRP alone. The AUC of CRP and PCT measurements together was significantly higher than the AUC of CRP alone (AUC of both CRP and PCT versus AUC of CRP alone: 0.858 versus 0.819 in all patients, *P* <0.001; 0.758 versus 0.684 for group I, *P* = 0.01; 0.899 versus 876 for group II, *P* = 0.01).

The best cutoff value for diagnosing infection in patients with impaired renal function (group II) was 3.08 mg/dL for CRP and 1.1 ng/mL for PCT. In patients with normal renal function, the best cutoff value was 2.49 mg/dL for CRP and 0.08 ng/mL for PCT.

**Figure 2:**
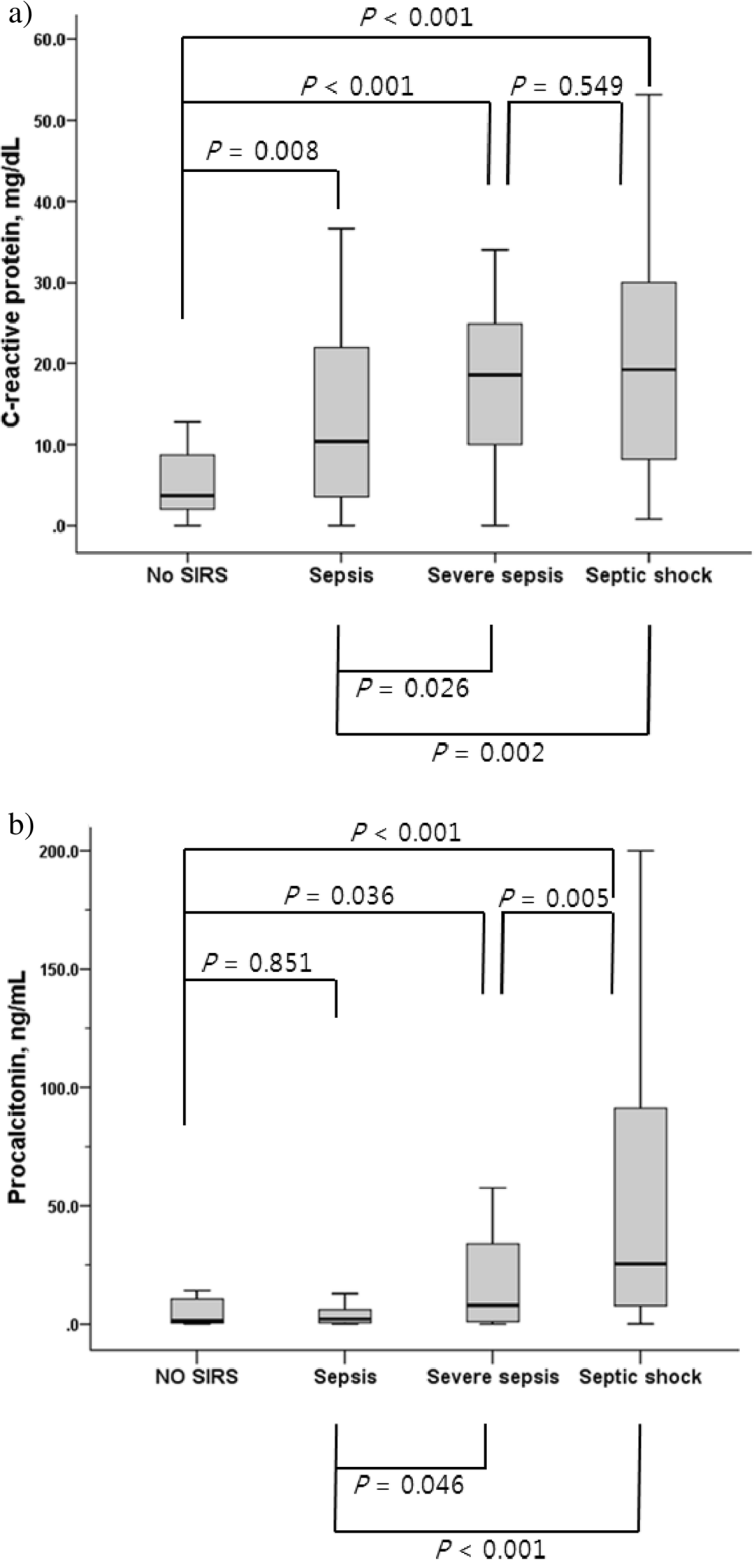
**The association between C-reactive protein (CRP) and procalcitonin (PCT) levels and the severity of infection in patients with impaired renal function.** The differences in CRP and PCT among four subgroups were analyzed using the Kruskal-Wallis test. **(a)** CRP increased depending on the infection severity (*P* <0.001). However, there was no difference in CRP between severe sepsis and septic shock (*P* = 0.549). The median (IQR) CRP in each subgroup was as follows: 3.74 (7.27) when there was no systemic inflammatory response syndrome (SIRS), 10.41 (18.82) in sepsis, 18.59 (15.43) in severe sepsis, and 19.26 (21.94) in septic shock. **(b)** PCT increased depending on the infection severity (*P* <0.001). However, PCT was not significantly higher in patients with sepsis than in those without SIRS (*P* = 0.851). The median (IQR) PCT in each subgroup was as follows: 1.52 (10.78) in the absence of SIRS, 2.22 (5.51) in sepsis, 7.96 (34.97) in severe sepsis, and 25.41 (85.11) in septic shock.

The association between CRP and PCT levels and the severity of infection in patients with impaired renal function (group II) was also analyzed. Group II patients with infectious diseases were categorized depending on the severity of their infections defined as no SIRS, sepsis, severe sepsis, or septic shock. Both CRP and PCT showed significant correlation with infection severity using Spearman correlation (CRP: *P* <0.001, *r* = 0.378; PCT: *P* <0.001, *r* = 0.414). The Kruskal-Wallis test was also performed to analyze the differences in CRP and PCT among four subgroups. There were differences in CRP and PCT among four subgroups (*P* <0.001 for both CRP and PCT) (Figure 2). However, the Tukey test using ranks for post-hoc comparison showed no significant differences in CRP between severe sepsis and septic shock (*P* = 0.549) (Figure 2a). In PCT, the Tukey test using ranks for post-hoc comparison showed no significant differences between absence of SIRS and sepsis (*P* = 0.851) (Figure 2b).

**Figure 3:**
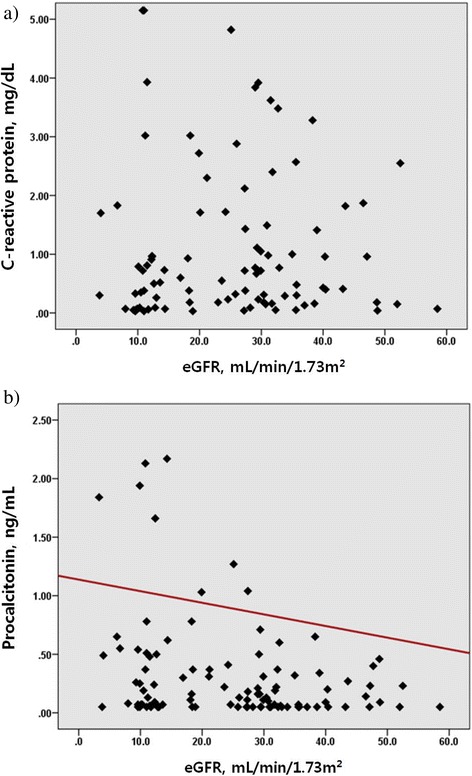
**Association between C-reactive protein (CRP) and procalcitonin (PCT) levels and estimated glomerular filtration rate (eGFR) in patients with impaired renal function.** Spearman correlation analysis was used to obtain *r*- and *P-*values. **(a)** CRP was not correlated with eGFR (*r* =0.037, *P* = 0.70). **(b)** PCT was significantly correlated with eGFR (*r* = −0.247, *P* = 0.01).

The correlation between CRP, PCT, and renal function was analyzed in patients without infection. Patients receiving renal replacement therapy were excluded in this analysis. CRP was not correlated with eGFR (*P =*0.70), whereas PCT was inversely correlated with eGFR (r = −0.247, *P* = 0.01) (Figure 3).

## Discussion

This study demonstrated that PCT is not superior to CRP for diagnostic accuracy, nor is it more reliably associated with infection severity considering renal function. CRP had diagnostic accuracy similar to PCT in patients with impaired renal function as well as in those with normal renal function. In patients with infection, CRP levels were higher in cases with less severe infection, which was a significantly different result than the levels observed among patients without SIRS, with sepsis, and with severe sepsis. PCT increased with more severe infection and there were no significant differences in PCT levels between patients with and without SIRS. Among patients without infection, CRP was not correlated with eGFR, while PCT was inversely correlated with eGFR.

A recent meta-analysis found that PCT was not superior to CRP in patients with impaired renal function [10]. In that study, hierarchical summary ROC curves had an AUC of 0.88 (95% CI 0.83, 0.92) for CRP and 0.89 (95% CI 0.86, 0.92) for PCT, which were similar to our results (AUC: 0.876, 95% CI 0.839, 0.912 for CRP; AUC: 0.876, 95% CI 0.839, 0.912 for PCT). Also in a recent study including a relatively smaller homogenous population of CKD patients, AUC was 0.860 (95% CI 0.712, 1.000) for CRP and 0.911 (95% CI 0.773, 1.000) for PCT. There was also no significant difference in AUC (*P* = 0.53, *z*-statistic = 0.62, 95% CI 0.110, 0.211) [15].

In addition to the finding that PCT is not superior to CRP, it is also more costly: PCT testing is eight times more expensive than CRP testing in Korea, and two to four times more expensive in the US and Europe. Among patients with normal renal function, our data showed higher sensitivity and specificity of PCT compared to CRP, which is consistent with the findings of previous studies [8,9]. Although the kinetics of PCT, including elimination route and mechanism, have not been fully elucidated, it is possible that impaired renal function or dialysis may influence PCT because of its low molecular weight (13 kDa) [16]. Previous studies that evaluated the influence of renal function or dialysis on PCT yielded inconsistent results [7,17-22]. Our study demonstrated a weakly significant correlation between PCT and eGFR in non-infected patients. This result implies that PCT may be also influenced by renal function. Furthermore, PCT was not distinctively elevated in patients with less severe infections, and the basal level of PCT increased in patients with impaired renal function [7,8,17-19,22]; thus, a higher cutoff value for PCT was suggested for use in patients with impaired renal function [7,10,18]. Similarly, in our study, the best cutoff values of PCT for diagnosing infection were 1.1 ng/mL for patients with impaired renal function and 0.08 ng/mL for patients with normal renal function.

Previous studies that investigated the association between infection severity and CRP or PCT did not specifically explore these relationships in patients with impaired renal function exclusively [8,23,24]. Still, those studies also found that CRP levels were higher in patients with mild organ dysfunction and sepsis, but CRP levels did not increase significantly with progression toward more severe stages of disease [8]. In contrast, PCT was reported to be modestly higher in cases of local infection and cases of infection without multiple organ failure [25]. Similarly, in our study, CRP levels were significantly different between impaired renal function cases without SIRS, with sepsis, and with severe sepsis. However, there was no significant difference between CRP levels in cases of severe sepsis and septic shock. In contrast with CRP results, PCT levels increased in patients with more severe organ dysfunction, severe sepsis, or septic shock. These differences are possibly due to different sources of CRP and PCT during the inflammatory process. CRP is produced only by *de novo* hepatic synthesis [6]. Therefore, CRP levels might not be increased further in patients with hepatic dysfunction, even under conditions of severe infection, such as septic shock or severe sepsis. Leukocytes are thought to be the source of PCT during sepsis, although there is still controversy regarding the origins of PCT [26]. During less severe infection, no SIRS, or sepsis, we observed a subtle increase in PCT levels, probably due to less specific stimuli to the leukocytes. These different responses, depending on the severity of infection, suggest that CRP would be a more valuable infection marker because CRP increases gradually, even in less severe infection, allowing for differential diagnosis of SIRS or no SIRS.

Interestingly, our study showed the best accuracy of CRP and PCT in patients with renal impairment. We compared the proportion of patients with septic shock with other subgroups (no SIRS, sepsis, and severe sepsis) using the chi-square test. The proportion of septic shock was greater in patients with renal impairment than those with normal renal function (the proportion of septic shock: 30% in renal impairment, 11% in normal renal function, *P* = 0.001). However, there was no difference in the overall severity of infection between renal impairment and normal renal function groups when analyzed by the Wilcoxon rank sum test (*P* = 0.133). Although the best accuracy of CRP and PCT in patients with renal impairment might be affected by the partial proportional difference of infection severity, we believe that this point did not substantially compromise the aim of this study to compare CRP and PCT as an infection marker.

This study has several limitations. First, there may have been some selection bias because of the retrospective design. Because there are no gold standard criteria for diagnosing infection, there might be some misclassifications of infection status in this study. However, the definition of infection has an inevitable methodological limitation in all similar studies. In order to overcome this limitation, all ambiguous cases were excluded from our study. Second, unexpected factors that could affect CRP and PCT levels were not concurrently analyzed in this study. However, cardiovascular disorder, a common factor known to increase CRP and PCT, was thoroughly analyzed alongside renal function, and no significant association was found between cardiovascular disease and eGFR (*P =* 0.64). Third, our study sample was not large. Nonetheless, we enrolled a comparable number of patients across each stage of renal function, including dialysis patients. Furthermore, to our knowledge, this study included the largest study population among single-center studies evaluating the diagnostic value of PCT in patients with impaired renal function, although the patient population of our study might be heterogeneous because of various hospitalization settings including wards, intensive care units, or emergency rooms. Nonetheless, as a single-center study, the measurement techniques and diagnostic criteria for infection used in our study were consistent across all participants.

## Conclusions

In conclusion, this study suggests that in spite of its higher cost, PCT is not superior to CRP as an infection marker in terms of diagnostic value. The sensitivity and specificity of PCT and CRP were comparable in our analyses; however, PCT levels had an inverse relationship with eGFR in patients with renal insufficiency but no infection. Finally, considering that PCT levels were not significantly different between patients without SIRS and patients with sepsis, CRP would be a more valuable marker of infection in those patients.

## Key messages

- Both CRP and PCT are useful to distinguish infectious conditions from non-infectious conditions, not only in patients with normal renal function, but also in patients with impaired renal function.
- Although the accuracy of CRP and PCT as infection markers was comparable in patients with impaired renal function, there was an inverse relationship between PCT and eGFR in patients without infection.
- PCT is not superior to CRP as an infection marker in terms of diagnostic value in patients with impaired renal function despite its higher cost.

## Abbreviations

AUC: areas under the respective curve
CKD: chronic kidney disease
CRP: C-reactive protein
DM: diabetes mellitus
eGFR: estimated glomerular filtration rate
HD: hemodialysis
HTN: hypertension
IQR: interquartile range
MDRD: modification of diet in renal disease
PCT: procalcitonin
PD: peritoneal dialysis
ROC: receiver operating characteristic
RRT: renal replacement therapy
SIRS: systemic inflammatory response syndrome
